# Reconstructing the interosseous ligament: A technique using a single bundle synthetic tape

**DOI:** 10.1016/j.jham.2026.100495

**Published:** 2026-08-10

**Authors:** Enrico Carità, Clelia Rota, Vasiliki Batsou, Mara Laterza, Alberto Donadelli

**Affiliations:** a Unità Operativa di Chirurgia della Mano e del Gomito, Clinica San Francesco, Verona, Italy; b Università degli Studi di Torino, Scuola di Specializzazione in Ortopedia e Traumatologia, Torino, Italy; c Department of Hand and Peripheral Nerve Surgery, CHR de la Citadelle, Liège, Belgium

**Keywords:** Essex-Lopresti injury, Interosseous membrane, Central band reconstruction, Forearm biomechanics

## Abstract

Reconstruction of the interosseous membrane of the forearm has gained popularity over recent decades and several surgical techniques have been described to treat the longitudinal instability of the forearm after an Essex-Lopresti injury (ELI). We propose a synthetic reconstruction of the interosseous ligament (IOL) with double Arthrex FiberTape® and present data from cadaveric specimens used to test the reconstruction. After reconstruction, proximal migratory forces were applied to the radius to assess the radioulnar displacement at wrist level. Safety of the technique was also assessed by measuring the distance between the fixation system and the posterior interosseous nerve (PIN). These experiments provide preliminary biomechanical data to support further evaluation of this technique for potential clinical application.

## 1 Introduction

The “forearm complex” concept, as defined by Soubeyrand and Dumontier in 2007 and 2011^1^^,^^2^ provides a framework for understanding forearm injuries. This concept states that the forearm is a single functional unit consisting of two bones, i.e. the radius and ulna, and the interosseous membrane (IOM); three joints are included in this functional unit: two anatomical joints (the distal and proximal radioulnar joints, respectively DRUJ and PRUJ), and one functional joint (the middle radioulnar joint, or MRUJ), which is made up of the forearm bones and the IOM. The three joints work together as “lockers”, i.e. as constraints, to provide forearm stability during pronation and supination.

The “Locker forearm theory”^3^ - the guideline for the management of forearm joints injuries - states that a constraint (or locker), alone, cannot provide forearm stability: when all constraints are disrupted (Essex-Lopresti Injury) at least two of them should be restored.

According to Heifner and Bain,^4^ in cases of acute or chronic ELI, despite the need for radial head replacement or fixation, central band (CB) repair provides most of the ligamentous stabilization and it is the first structure addressed to restore the anatomic position of the proximal radius and the radioulnar relationship. Then, the radial head is replaced or fixed to provide the necessary osseous integrity and stability. The authors state that further DRUJ stabilization is not immediately necessary. We fully agree with Heifner and Bain^4^ that a difference of 2–3 mm in ulnar variance compared with the contralateral side represents the threshold for considering reconstruction of the central band. As highlighted in the work by McGinley et al.,^5^ the central band, owing to its low elastin content and intrinsic stiffness, is likely to be disrupted when proximal radial migration exceeds 2 mm.

Numerous methods of IOL reconstruction have been described, differing in the type of the graft, number of bundles and fixation, without demonstration of significant superiority. According to the study of Rein et al., in 2020,^6^ the central band or interosseous ligament (IOL) is not as densely innervated as the rest of the membrane, which correlates with its static stability role; reconstruction of CB can be successfully performed by non-sensory tissue (flexor carpi radialis, palmaris longus, semitendinosus) or material.

The present study describes a surgical technique for interosseous ligament reconstruction using a double synthetic graft (Arthrex FiberTape®) fixed with two SwiveLock® screws and evaluates its biomechanical contribution to resistance against proximal radial migration under controlled axial loading, providing a rationale for further biomechanical evaluation and eventual clinical investigation in acute and chronic Essex-Lopresti injuries.

## 2 Theory

This biomechanical forearm specimen model was designed to examine the mechanical role of the reconstructed central band under controlled axial loading. A sequential sectioning protocol was used to progressively remove native longitudinal stabilizers while maintaining the reconstructed interosseous ligament in situ, allowing assessment of changes in proximal radial migration attributable to each condition. The model specifically evaluates resistance to proximal migration under static, monotonic loading and does not address cyclic behavior or clinical outcomes.

## 3 Materials and methods

### 3.1 Setup

We performed a biomechanical study on eight fresh-frozen cadaveric upper limbs to test the surgical technique of using synthetic Arthrex FiberTape® and Arthrex SwiveLock® screws to reconstruct the central band. Based on the study of Masouros et al.^7^ and Farr et al.,^8^ we considered our sample size to be appropriate for the specific objectives of the present study. According to institutional policy, formal institutional review board approval was not required because no living human subjects or identifiable private information were involved. Donor age and sex were not available in the de-identified specimen records provided to the authors.

Neutral ulnar variance, assessed fluoroscopically using a C-arm with the forearm positioned in neutral rotation, was a specific inclusion criterion for specimen selection. Specimens showing degenerative changes at the distal radioulnar joint or at the proximal radioulnar and radiocapitellar joints at the elbow, as well as those with evidence of previous traumatic injury, were excluded from the study.

All reconstructions were performed by the same experienced surgeon according to the new technique.

### 3.2 Superficial and deep exposure

The bony contours and the course of the interosseous ligament (IOL) were outlined on the skin according to the anatomical descriptions reported by Bin Abd Razak et al.^9^ Skin marking was performed with the forearm in full supination, a position in which IOL strain is minimal.^10^^,^^11^

**Fig. 1:**
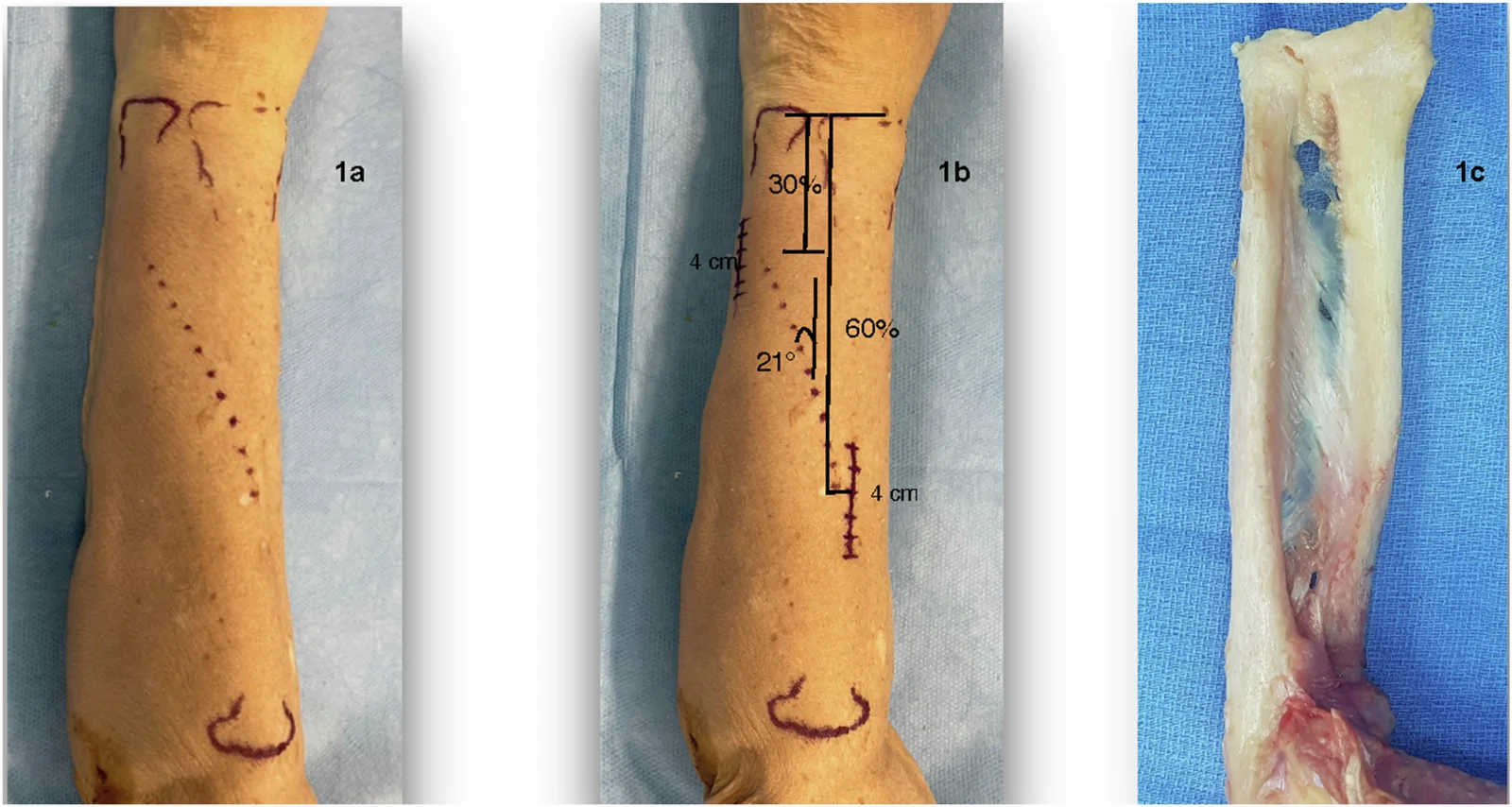
**(a–c)**: Marking of anatomical landmarks on the cadaveric specimens. 1a: Skin marking of bony contours and planned IOL course. 1b: Planned reconstruction landmarks and skin incisions. 1c: Anatomical exposure of the interosseous membrane.

The ulnar insertion of the IOL is broader and located at the junction between the middle two thirds and the distal one third of the ulna.^12^ The radial origin is slightly narrower and is located at 60% of the radial length measured from the styloid^13^ (Fig. 1b).

An approximately 4-cm longitudinal incision centered over the planned radial insertion site was performed. The interval between the brachioradialis and the extensor carpi radialis longus was developed just proximal to the pronator teres, and blunt muscle splitting was used to expose the radial shaft while carefully protecting the superficial sensory branches of the radial nerve and the posterior interosseous nerve.

For distal ulnar exposure, a second approximately 4-cm longitudinal incision was made at the level of the planned bone tunnel exit, with the forearm maintained in neutral rotation. The interval between the flexor carpi ulnaris and the extensor carpi ulnaris was then developed to access the ulna.

### 3.3 Reconstruction

Bone tunnels were created with the forearm in an intermediate prono–supination position, to ensure their emergence along the medial and lateral crests of the radius and ulna, respectively, corresponding to the native insertion sites of the interosseous membrane.

**Fig. 2:**
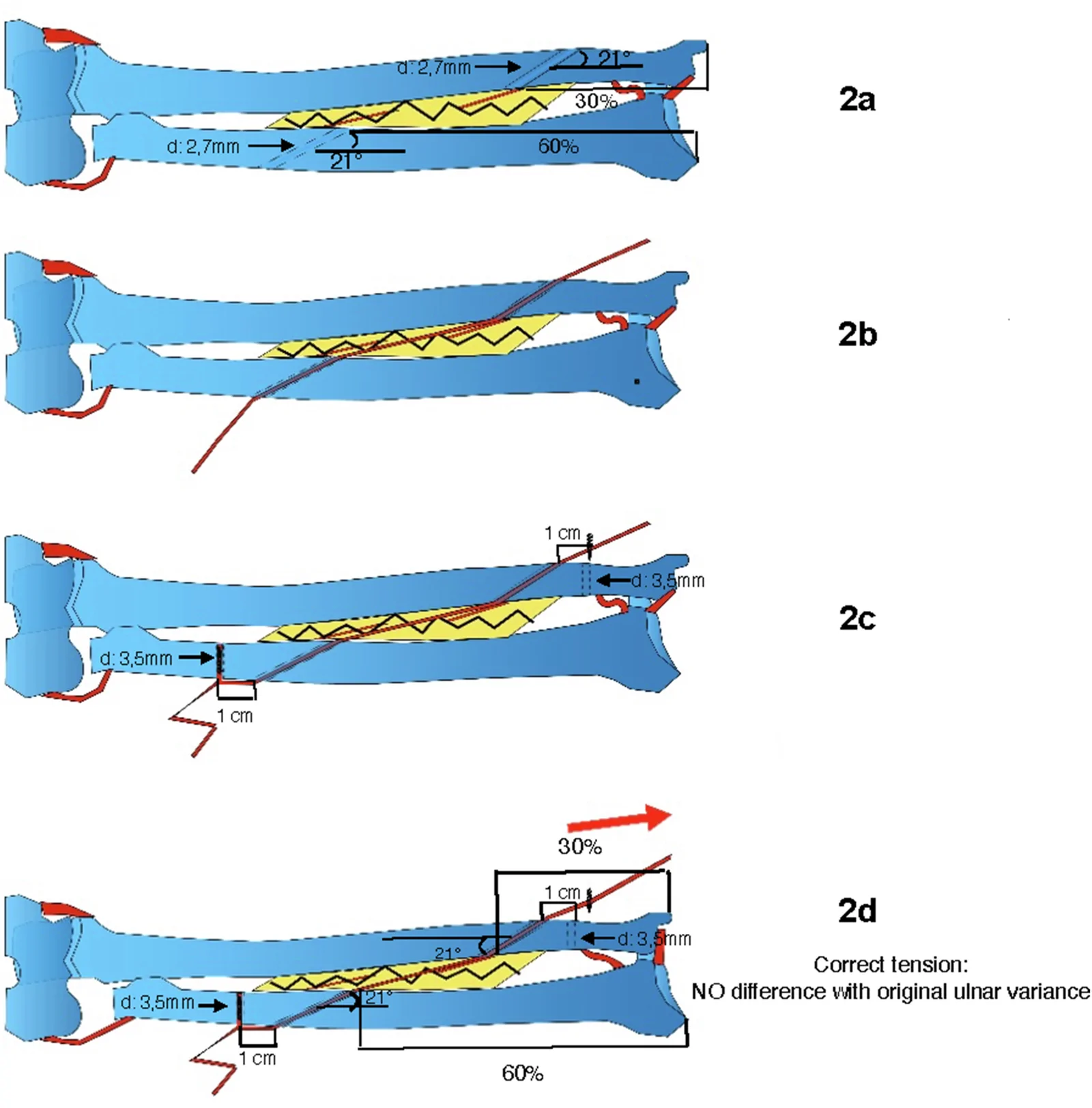
**(a–d)**: Schematic illustration of the surgical technique. 2a: Graft bone tunnels. 2b: Passing the double FiberTape®. 2c: SwiveLock® anchor drill holes. First, the double tape is fixed to the radius. 2d: The double tape is fixed to the ulna while the radius is pulled distally.

During tunnel creation, careful attention was paid to reproduce the direction and angulation of the central band, i.e. 21°, as described in the studies by Noda et al.^14^ and Farr et al.^8^ Accordingly, a 2.7 mm diameter bone tunnel was first drilled in the radius at an angulation of approximately 21° (Fig. 2a). A second 2.7 mm diameter bone tunnel was then drilled in the ulna, maintaining the same 21° orientation. Particular attention was paid to ensuring that the two bone tunnels were strictly coaxial.

**Fig. 3:**
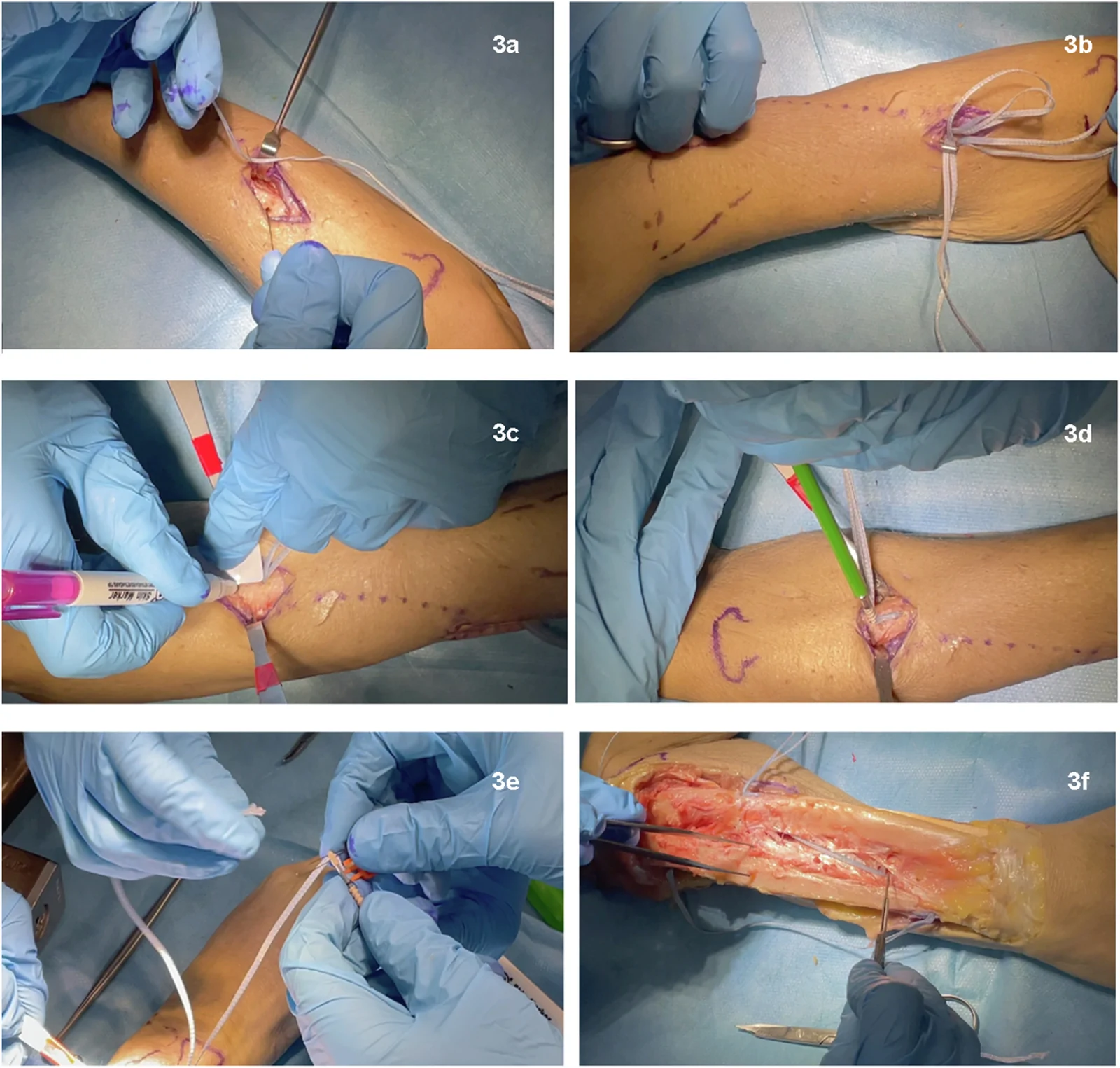
**(a–f)**: Surgical technique in cadaveric specimens. 3a: Passage of the double FiberTape® through the radial bone tunnel. 3b: Passage of the double FiberTape® along the dorsal aspect of the native interosseous membrane. 3c: Identification of the radial entry point for creation of the drill hole for SwiveLock® anchor insertion, approximately 1 cm proximal to the FiberTape® tunnel. 3d: Radial fixation of the double FiberTape® using a SwiveLock® anchor. 3e: Double FiberTape® loaded into a SwiveLock® anchor for distal ulnar fixation. 3f: Removal of dorsal forearm muscles and tendons to visualize the construct.

Taking into account the skin landmarks, double FiberTape® (Arthrex, Naples, FL, donated by the company to our institution) was first introduced through the radial bone tunnel using a metallic suture-pass (Fig. 3a), then a smooth instrument like a right Kocher or a Pennington was passed on the dorsal side of the native IOL, under extensor muscles, from the distal ulnar access to the proximal radial one, to retrieve the tape according to the planned direction of the new IOL (Figs. 2b and 3b). Potential risks include soft-tissue entrapment or conflict (muscle bellies, extensor tendons and the anterior and posterior interosseous nerves): these were assessed by repeatedly sliding the tape to confirm smooth passage without resistance. Furthermore, extensor tendon inclusion can be checked and excluded by testing tendon gliding and finger flexion/extension during tape sliding.

Finally, the double tape was passed into the ulnar bone tunnel again using the metallic suture-pass.

Two bone fixation tunnels of 3.5 mm were drilled afterwards to insert the SwiveLock® screws (also donated by the company to our institution) with proper instrumentation, the radial one 1 cm proximal and the ulnar one 1 cm distal to the respective bone tunnels (Figs. 2c and 3c).

To reduce the “saw effect” on the bone tunnel edges by the FiberTape®, the direction of the screw tunnels must diverge at an angle of approximately 30–40° from the direction of the graft tunnels.

Furthermore, oblique screw tunnels provide two additional advantages: they allow graft tensioning during screw insertion in the same direction as the applied graft traction and reduce the risk of prominence on the radial side of the ulna, particularly in thin bone.

The double FiberTape® was first fixed proximally to the radius with a SwiveLock® anchor (Fig. 3d) and then manually tensioned with the forearm in maximal supination. This position represents the shortest distance between the radius and ulna and allows maximal graft tensioning, as reported by Nakamura et al.^10^ and Farr et al.^8^

**Fig. 4:**
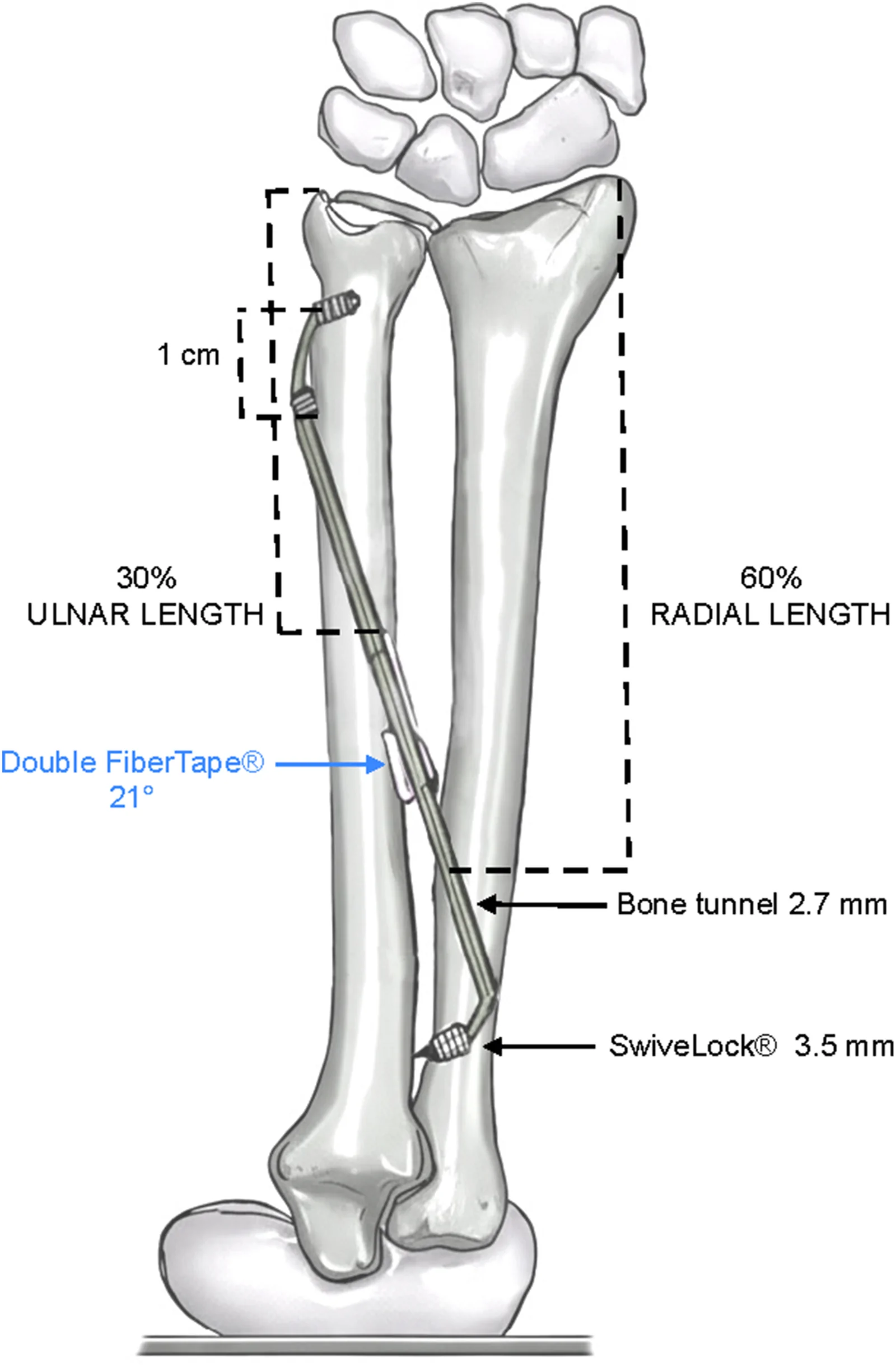
Schematic representation of the proposed construct.

When the desired tension was achieved (no variation of original ulnar variance, reassessed fluoroscopically), the distal screw on the ulnar side was driven into the hole according to the manufacturer's instructions to generate and maintain the expected tension (Figs. 2d and 3e). The final construct is shown schematically in Fig. 4.

In any case, because the procedure was simulated in specimens with an intact interosseous membrane and preserved distal radioulnar and elbow joints, the reconstruction itself was considered unlikely to alter ulnar variance.

Before introducing the SwiveLock® screw, in case of small bones, reduction of excursion between the interference screw and the top of the anchor may prevent breach of the second cortex, especially in small distal ulna. Pronation and supination were checked at the end of the procedure.

### 3.4 Procedure completion and outcome measurement

**Fig. 5:**
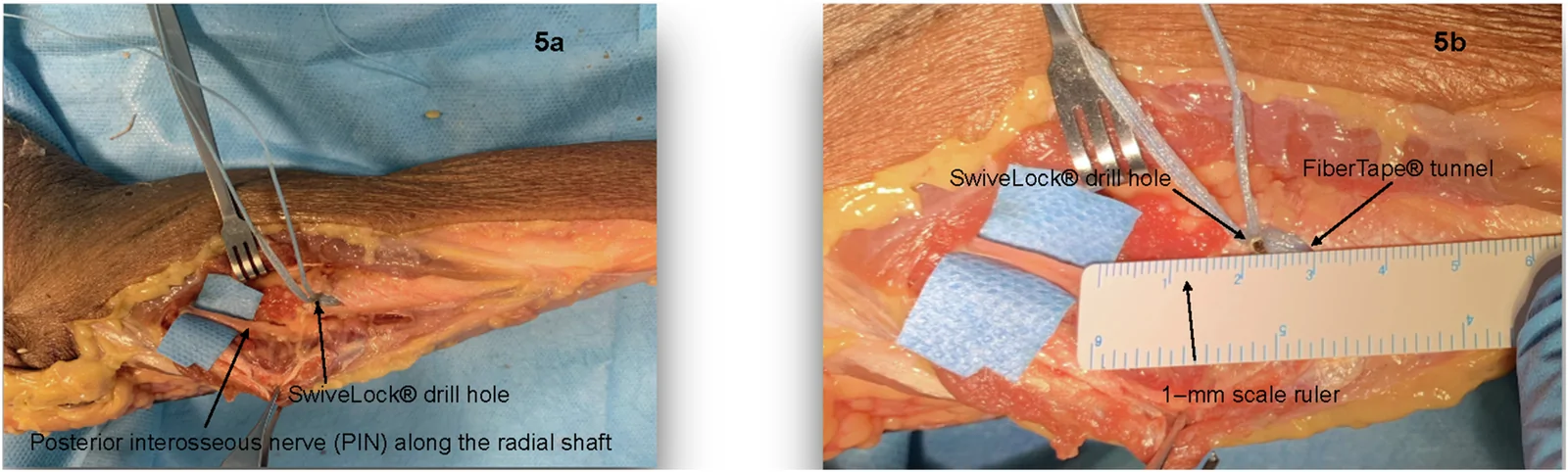
**(a–b)**: Shortest distance between the SwiveLock® drill hole and the posterior interosseous nerve (PIN) along the radial shaft, measured using a millimeter ruler. 5a: Posterior interosseous nerve (PIN) exposed along the radial shaft, with the SwiveLock® drill hole visible. 5b: Measurement of the shortest distance between the SwiveLock® drill hole and the posterior interosseous nerve using a millimeter ruler. The FiberTape® tunnel is also shown.

At the end of the procedure, all muscular and tendinous structures of the dorsal forearm were removed to verify that the reconstructed central band tension was not limited by soft-tissue interposition and to visually confirm reproduction of the native interosseous ligament course (Fig. 3f). The posterior interosseous nerve (PIN) was dissected and the shortest distance between the radial SwiveLock® screw drill hole and the nerve along the radial shaft surface was measured using a ruler with 1-mm accuracy (Fig. 5a and b).

The specimens were subsequently mounted vertically on a servo-hydraulic materials testing system (MTS, Eden Prairie, MN), equipped with an integrated load cell and linear displacement transducer, capable of generating a maximum axial load of 250 N. The testing apparatus allowed precise control of axial loading conditions while continuously recording force and actuator displacement throughout the experiment.

**Fig. 6:**
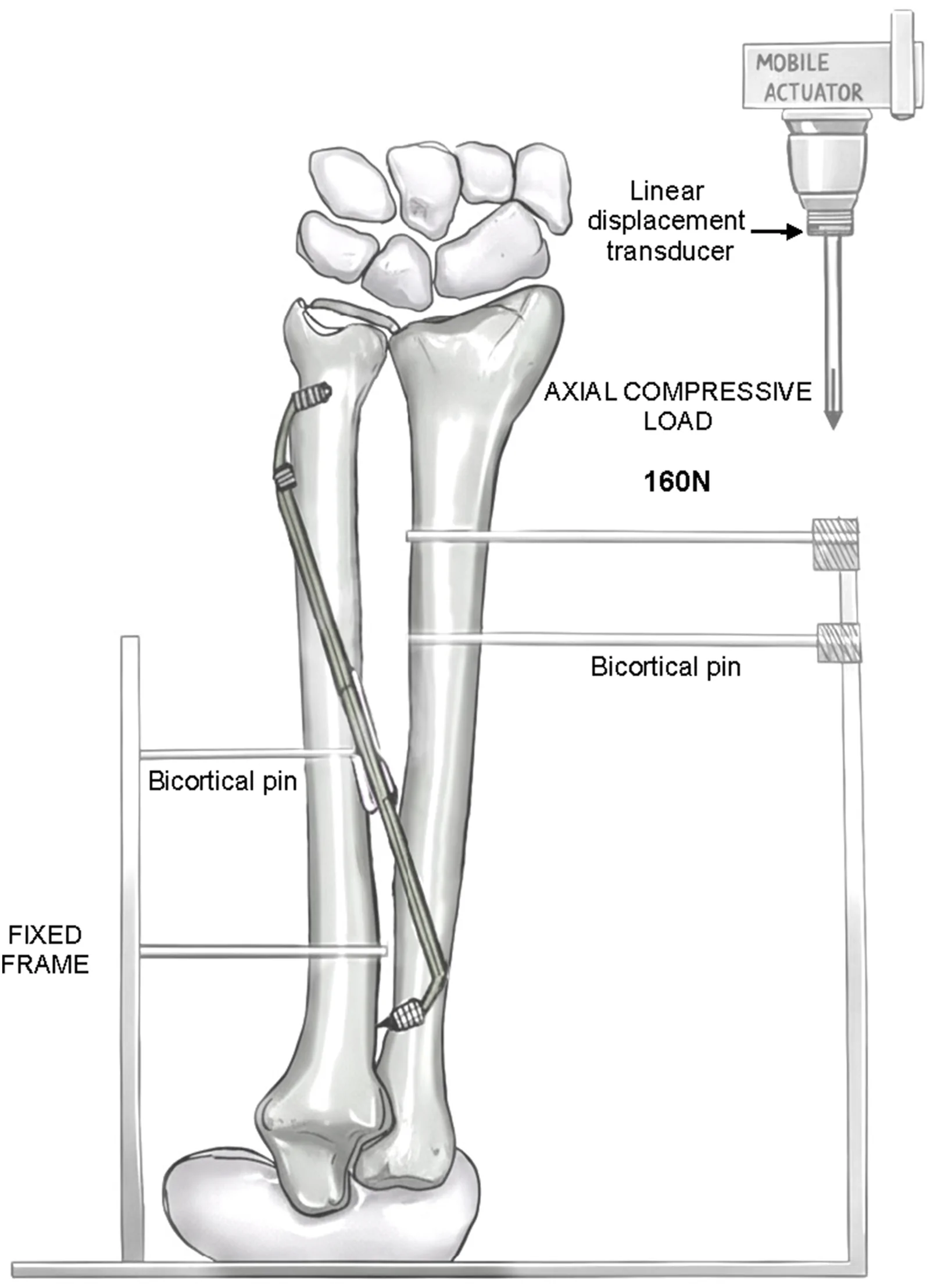
Schematic illustration of the experimental setup and pin configuration used for axial loading of the radius. Radial half-pins were connected to the mobile actuator while ulnar half-pins were fixed to the frame, ensuring isolated axial loading and independent longitudinal radial motion.

Two half-pins were inserted bicortically into the radial shaft and rigidly connected to the mobile actuator of the testing system, which applied axial compressive load exclusively to the radius. Two additional half-pins were inserted into the ulnar shaft and fixed to the stationary frame of the device, thereby providing a stable reference and preventing ulnar motion during testing. None of the pins penetrated the other bone, ensuring complete mechanical independence between the radius and ulna (Fig. 6).

This configuration enabled isolated assessment of proximal radial migration under axial loading, with longitudinal translation of the radius measured directly from the displacement data recorded by the MTS system. Reference marks were drawn on the cortical surface of the radius and ulna at the level of the distal radioulnar joint to document the experimental setup during sequential testing.

Radial displacement was used as the primary outcome measure. Axial loading was applied collinearly with the longitudinal axis of the radius using a servo-hydraulic testing system (MTS) operating in controlled monotonic mode.

A baseline measurement was first obtained with all native stabilizers preserved and the double FiberTape® central band reconstruction in place. Subsequently, all specimens were tested sequentially under four progressive conditions: (1) after excision of the radial head, (2) after sectioning of the native interosseous ligament, (3) after additional sectioning of the triangular fibrocartilage complex insertion, and (4) after sectioning of the reconstructed interosseous ligament. For each testing condition, axial force was progressively increased until reaching 160 N, a reproducible sub-failure axial load commonly used to assess longitudinal forearm stability in several cadaveric models,^15^^,^^16^ while minimizing the risk of permanent structural damage to the specimens. Once the target load was achieved and maintained by the testing system, the MTS displacement signal was allowed to reach a stable plateau before proximal radial migration was recorded. Axial displacement measured directly by the MTS actuator served as the sole quantitative parameter for assessment of longitudinal translation, assuming rigid ulnar fixation and constrained axial loading as commonly adopted in biomechanical forearm testing. For each condition, axial load was applied once in a single monotonic sequence. The entire loading protocol—including load application rate, stabilization time at the target load, and the interval between successive testing conditions—was identical across all eight specimens and all tested configurations, ensuring methodological consistency and comparability.

After completion of displacement measurements at 160 N in the fourth testing condition with the FiberTape® reconstruction still intact, and prior to its sectioning, axial load was further increased once per specimen, in a standardized manner across all specimens, to a maximum of 250 N, corresponding to the intrinsic load limit of the testing system, solely to verify construct integrity and exclude gross failure or fixation loosening; this higher load was not used for displacement analysis. The experimental design intentionally focused on controlled monotonic axial loading to determine whether the proposed central band reconstruction limits proximal radial migration under controlled sub-failure axial loads (160 N). Within this framework, construct failure was defined as macroscopic graft rupture, fixation failure (screw pull-out or mobilization), or an abrupt uncontrolled increase in radial displacement indicating loss of longitudinal constraint. Time-dependent phenomena such as creep, tunnel enlargement, progressive loss of reduction, and fatigue-related fixation changes were beyond the scope of the present model, as they require dedicated cyclic loading protocols to assess fatigue behavior and viscoelastic response.

### 3.5 Statistical analysis

All statistical analyses were performed using a repeated-measures design, as each specimen underwent baseline evaluation followed by all sequential experimental conditions. Owing to the limited sample size and the non-normal distribution typically observed in biomechanical displacement data, nonparametric methods were selected a priori.

Overall differences among testing states were assessed using the Friedman test for related samples. When a significant global effect was detected, post hoc comparisons between R-IOL-SEC and each preceding condition were performed using Wilcoxon signed-rank tests with Bonferroni adjustment for four comparisons. Statistical significance was set at α = 0.05; Bonferroni-adjusted thresholds were applied to post hoc comparisons.

Data are presented as mean ± standard deviation for descriptive reporting, whereas statistical inference was based on rank statistics. All measurements were obtained using a standardized acquisition protocol within the same experimental session under controlled laboratory conditions. Interobserver reliability analysis was not performed because measurements were acquired directly from the MTS transducer within a single standardized experimental session. No formal a priori power analysis was performed due to the absence of preliminary data suitable for sample size estimation in this experimental model; therefore, each specimen served as its own matched control to maximize statistical efficiency. Analyses were performed using SPSS software (IBM Corp., Armonk, NY, USA).

## 4 Results

The reconstruction procedure was completed in all specimens without iatrogenic injury or soft-tissue interposition. The reconstructed central band followed the native anatomical course in all cases.

Proximal radial migration under axial loading for each testing condition is reported in Table 1. Data are presented as mean ± standard deviation (SD). In the intact configuration (INT), mean migration was 0.7 ± 0.8 mm. After radial head excision (RH-EX), migration was 0.8 ± 0.8 mm. Sectioning of the native interosseous ligament (IOL-SEC) increased migration to 1.4 ± 0.6 mm, and additional sectioning of TFCC (TFCC-SEC) increased migration to 2.1 ± 0.8 mm. Following sectioning of the reconstructed interosseous ligament (R-IOL-SEC), migration increased to 10.9 ± 3.5 mm.

**Table 1:** Proximal radial migration (mm) recorded in each specimen under sequential experimental conditions.

Specimen	INT	RH-EX	IOL-SEC	TFCC-SEC	R-IOL-SEC
1	0	0.5	1	1.5	8
2	1	1	1.5	2.5	13
3	0	0	0.5	1	7
4	1.5	1.5	2	3	8
5	0	0	2	2.5	11
6	0	0	0.5	1.5	9
7	2	2	2	3	17
8	1	1	1.5	1.5	14
Mean	0.7	0.8	1.4	2.1	10.9
SD	0.8	0.8	0.6	0.8	3.5

Note: INT = all native stabilizing structures intact; RH-EX = radial head excision; IOL-SEC = sectioning of the native interosseous ligament; TFCC-SEC = sectioning of the triangular fibrocartilage complex; R-IOL-SEC =sectioning of the reconstructed interosseous ligament.

Because measurements were repeated within the same specimens across testing conditions, a nonparametric repeated-measures analysis (Friedman test) was performed. This demonstrated a significant overall effect of testing condition on proximal radial migration (p < 0.001). Post hoc pairwise comparisons demonstrated significantly greater migration after sectioning of the reconstructed interosseous ligament (R-IOL-SEC) compared with all preceding testing conditions (Bonferroni-adjusted p < 0.05).

No construct failure, according to the predefined criteria, occurred during axial loading up to 250 N, as no macroscopic graft rupture, screw pull-out or mobilization, or abrupt loss of longitudinal constraint occurred.

The mean shortest distance between the radial SwiveLock® screw drill hole and the posterior interosseous nerve was 14 mm (range, 11–16 mm) (Fig. 5a and b).

## 5 Discussion

The reported technique may represent a reproducible method for IOL reconstruction through limited surgical exposure, while following the native anatomical course of the interosseous ligament.

The primary stabilizing component of the forearm is the radial head, which restrains the radius from proximal migration. If the radial head is absent or insufficient, the central band (CB) of the IOM and the triangular fibrocartilage complex (TFCC) serve as the primary restraints^17^^,^^18^ limiting proximal radial migration. In their mechanical analysis, Hotchkiss et al.^19^ found that the TFCC accounts for 8% of the longitudinal forearm stiffness following radial head excision, whereas the CB accounts for 71%. This explains why we focused on CB reconstruction to provide biomechanical support for its eventual clinical application in forearm longitudinal instability.

Following injury, reconstruction is preferred over repair, as pointed out by Adams^13^: tears are typically intrasubstance and the interposition of muscles makes apposition of the thin CB difficult, particularly in view of its high collagen-to-elastin ratio and limited elastic behavior.^7^ When muscle interposition or herniation is present, predictable direct healing of the native ligament is unlikely.

After the first attempt at IOM reconstruction in 1995, performed by Sellman^20^ with a braided polyester cord, several grafts have been proposed by different authors: bone-patellar tendon-bone grafts (Gaspar et al., 2018),^21^ tibialis anterior (Miller et al., 2016),^22^ Achilles tendon (Stabile et al., 2005),^23^ semitendinosus tendon (Soubeyrand et al., 2006),^24^ palmaris longus (Tejwani et al., 2005),^16^ fascia lata (Bigazzi et al., 2017),^25^ flexor carpi radialis (Skahen et al., 1997),^17^ rerouting the pronator teres tendon (Chloros et al., 2008),^26^ synthetic grafts (Sabo and Watts, 2012)^27^ and TightRope® tenodesis (Brin et al., 2014).^28^

Within this reconstructive framework, the FiberTape®/SwiveLock® construct was not conceived as an internal brace whose effect would depend on predictable healing of the native central band. Rather, it was designed as a synthetic reconstruction intended to reproduce its static stabilizing role and to resist proximal radial migration under controlled axial loading.

The use of double FiberTape® with SwiveLock® anchors may offer several technical advantages. The diameter of the bone tunnels required for FiberTape® is smaller than that used in reconstructions with other grafts, which may limit local stress concentration and structural weakening when tunnels are correctly centered in the forearm bones.

Moreover, the construct is designed to transmit forces along the direction of graft tension rather than through opposing cortical buttons, which may reduce metal-to-bone friction at the cortical surface.

The SwiveLock® anchor allows a double fixation of the graft, one at the top of the screw, enabling precise adjustment of the desired graft tension, and a second along two sides of the screw body itself, functioning as an interference screw by pulling rather than pushing the graft, which may help reduce the risk of graft slippage and detensioning. Placement of the fixation system separate from the FiberTape® bone tunnels works like a hook, dissipating traction forces to the bone and potentially reducing the risk of bone resorption and fractures related to metallic fixation elements, while allowing the graft to be secured in the same direction of tensioning during screw insertion. This differs from traditional interference screw fixation, which works in the opposite direction.

The 3.5 mm diameter tunnel is completely filled by the SwiveLock® screw, eliminating frictional elements; therefore, forces are transmitted primarily within the tunnel and along its axis, without levering on the medial or lateral cortex (as occurs with the use of an endobutton).

The use of a double FiberTape® construct is intended to provide a potential secondary restraint in case of loosening or loss of tension of one tape. However, this protective effect under repetitive loading was not evaluated in the present study.

### 5.1 Our results

Final inspection showed that the reconstructed band followed the expected anatomical course without soft-tissue interposition or tunnel-related fractures.

Under controlled monotonic axial loading, proximal radial migration across the sequential destabilization stages remained comparable while the FiberTape® central band was intact; repeated-measures analysis demonstrated a significant increase only after sectioning of the reconstructed band.

No graft rupture or fixation loosening occurred during loading up to 250 N. This finding indicates preservation of gross construct integrity within the limits of the single-cycle monotonic loading protocol.

The synthetic construct avoids graft harvesting and related scarring or morbidity and allows a standardized reconstruction through limited surgical exposure.

### 5.2 Technical considerations

Potential technical considerations should be taken into account during central band reconstruction. Eccentric bone tunnel placement may predispose to intraoperative or postoperative fracture, for which plate fixation is indicated. Particular attention must therefore be paid to maintaining centered drilling in both bones.

Coaxial alignment of the graft tunnels reduces shear stress on the FiberTape®, which could otherwise weaken the graft and decrease construct tension.

The only step that may influence construct behavior is distal fixation tensioning. Proper use of the SwiveLock® anchor and appropriate graft tension are therefore essential. Inadequate planning of graft tension before placement of the distal screw may result in excessive tightness or laxity.

Although not evaluated in the present biomechanical model, during surgery it could be important to pre-test graft tension while verifying correct DRUJ alignment under fluoroscopy. A temporary K-wire fixing the distal radius at the appropriate height can be an option to maintain the correct ulnar variance before distal fixation. Use of a wrist arthroscopy traction tower can be another option to achieve correct DRUJ alignment.

In case of unsatisfactory tensioning, the SwiveLock® anchor can be removed and repositioned if the anchor tip with crossing FiberTape® can be retrieved. Inadequate pre-drilling or FiberTape® interposition may hinder proper seating of the interference screw, in which case trimming of the tape may be required to allow complete insertion.

### 5.3 Limitations of the study

This biomechanical model was designed as an initial controlled evaluation of the ability of the reconstructed central band to resist proximal radial migration under monotonic axial loading conditions. It does not replicate the complex mechanical environment associated with daily functional activities, including forearm rotation and muscular forces, which require further dedicated testing protocols.

Given the central band's role in repetitive axial load transmission, any reconstruction intended to reproduce its function requires assessment of time-dependent behavior—including fatigue response, creep, progressive elongation, and maintenance of graft tension—through dedicated cyclic loading protocols. In this context, progressive elongation or loss of graft tension may impair the longitudinal load-transmission function of the reconstructed interosseous membrane and may be more relevant than acute rupture when considering eventual clinical translation. This consideration may be particularly relevant for purely synthetic reconstructions, in which maintenance of tension over time occurs without biological remodeling of the graft, whereas biological incorporation may theoretically contribute to long-term maintenance of tension. However, the available literature remains limited and heterogeneous. Mid- to long-term clinical follow-up has been reported after both biological graft procedures, including bone-patellar tendon-bone graft reconstruction with mean follow-up greater than 10 years,^21^ and synthetic techniques, including synthetic braided cross-linked graft reconstruction with median follow-up of 8.5 years.^29^ Given the limited number of studies and the heterogeneity in study design, patient selection, follow-up, and reconstructive techniques, the available evidence does not currently allow a definitive conclusion regarding the superiority of either biological or synthetic reconstruction.

The technique was tested in specimens with an intact native interosseous membrane (IOM), which likely facilitated passage of the double FiberTape® along the dorsal aspect of the forearm; accordingly, no cases of soft-tissue entrapment were observed. In the setting of IOM disruption, identification of the volar and dorsal planes may be more challenging, with a potential risk of including adjacent soft tissues such as muscle bellies, extensor tendons, or the anterior interosseous nerve (AIN) and posterior interosseous nerve (PIN) during graft passage. Tendon inclusion can be recognized by intraoperative assessment of tendon gliding prior to distal fixation, whereas involvement of the AIN or PIN at this level could have consequences that were not assessed in this experimental model. In the clinical setting of Essex-Lopresti injury, which is associated with high-energy trauma, muscle herniation may be present in some cases^30^; when present, it may further limit any potential contribution of native tissue healing and complicate identification of the interosseous membrane plane, graft passage, and tensioning. This aspect could not be evaluated in the present cadaveric model and should be carefully assessed before any potential clinical application.

Furthermore, because reconstruction was performed before the forearm became longitudinally unstable, tensioning of the FiberTape® did not require restoration of ulnar variance, which would typically be necessary in a true Essex-Lopresti condition.

Additional limitations include the small number of specimens and the absence of clinical follow-up. Future studies should include dedicated cyclic biomechanical testing to evaluate time-dependent behavior and clinical investigation to determine in vivo applicability and outcomes.

## 6 Conclusions

After an Essex-Lopresti injury, restoration of at least two of the three forearm stabilizing units is required to limit proximal radial migration. Given its biomechanical role, interosseous membrane reconstruction should therefore be considered in addition to PRUJ repair.

The described technique is not technically demanding and may represent a reproducible option for central band reconstruction in the setting of Essex-Lopresti injury, especially when combined with procedures addressing the other lockers.

In this cadaveric controlled monotonic axial loading model, the proposed reconstruction—characterized by double graft fixation on the top of the screw and on the screw within separate bone tunnels—maintained limited proximal radial migration while intact, with a significant increase occurring only after sectioning. Furthermore, the technique is performed through limited surgical exposures and avoids autologous graft harvest.

These findings represent preliminary static biomechanical evidence under the tested conditions. Further investigations using cyclic loading protocols are required to evaluate time-dependent behavior, and clinical studies are needed to determine in vivo applicability and outcomes.

## Declaration of competing interests

The authors declare the following financial interests/personal relationships which may be considered as potential competing interests: Enrico Carità reports that equipment, drugs, or supplies were provided by Arthrex Italia S.R.L. The other authors declare that they have no known competing financial interests or personal relationships that could have appeared to influence the work reported in this paper.
